# Aryl Hydrocarbon Receptor Regulates Apoptosis and Inflammation in a Murine Model of Experimental Autoimmune Uveitis

**DOI:** 10.3389/fimmu.2018.01713

**Published:** 2018-07-25

**Authors:** Yike Huang, Junchi He, Huaping Liang, Ke Hu, Shaoqiu Jiang, Lu Yang, Suyin Mei, Xiao Zhu, Jing Yu, Aize Kijlstra, Peizeng Yang, Shengping Hou

**Affiliations:** ^1^The First Affiliated Hospital of Chongqing Medical University, Chongqing Eye Institute, Chongqing Key Laboratory of Ophthalmology, Chongqing, China; ^2^State Key Laboratory of Trauma, Burns and Combined Injury, Research Institute of Surgery, Daping Hospital, Third Military Medical University, Chongqing, China; ^3^Guangdong Provincial Key Laboratory of Medical Molecular Diagnostics, Dongguan Scientific Research Center, Guangdong Medical University, Dongguan, China; ^4^University Eye Clinic Maastricht, Maastricht, Netherlands

**Keywords:** TCDD, aryl hydrocarbon receptor, experimental autoimmune uveitis, blood–retinal barrier, macrophage/microglia, apoptosis

## Abstract

Uveitis is characterized as a common cause of blindness worldwide. Aryl hydrocarbon receptor (AhR), a ligand-activated nuclear receptor, has been implicated to play a role in human uveitis, although the exact mechanisms remain poorly understood. The purpose of this study was to enhance our knowledge concerning the role of AhR during intraocular inflammation. We immunized wild-type and AhR-knockout C57BL/6J mice with IRBP_651–670_ to induce experimental autoimmune uveitis (EAU). Disease severity was evaluated with both clinical and histopathological grading. Blood–retinal barrier (BRB) integrity was tested by Evans blue and tight junction proteins qualifications. Apoptosis was measured using TdT-mediated dUTP nick end labeling staining. Macrophage/microglia activation and polarization were studied by immunofluorescence and Western blot. Following EAU induction, AhR^−/−^ mice had more severe clinical and histopathological manifestations of uveitis than AhR^+/+^ mice. Increased vascular permeability and apoptotic cells were observed in AhR^−/−^ EAU mice when compared with AhR^+/+^ EAU mice. In addition, AhR^−/−^ EAU mice showed evidence of a significantly increased macrophage/microglia cells and a stronger polarization from the M2 to the M1 phenotype as compared to AhR^+/+^ EAU mice. The levels of pro-inflammatory cytokines including tumor necrosis factor-α (TNF-α), interleukin (IL)-6, and IL-1β were increased in AhR^−/−^ EAU mice, which was associated with the activation of NF-κB and signal transducers and activators of transcription (STAT) pathways. 2,3,7,8-tetrachlorodibenzo-p-dioxin (TCDD), an agonist of AhR, caused a significant decrease in the clinical and histopathological manifestations, preserved BRB integrity, reduced apoptotic cells, inhibited macrophage/microglia activation, and shifted their polarization from M1 toward M2. Moreover, decreased expression of pro-inflammatory cytokines including TNF-α, IL-6, and IL-1β and inhibition of NF-κB and STAT pathways were found in EAU mice following TCDD treatment. In conclusion, AhR activation with TCDD exhibits an immunomodulatory effect by reducing BRB breakdown, inhibiting retinal cell apoptosis, and reducing pro-inflammatory cytokine expression during EAU. The underlying mechanism may involve the modulation of macrophages/microglia polarization and the downregulation of NF-κB and STAT pathways.

## Introduction

Uveitis, a vision-threatening intraocular inflammatory disease, usually affects people aged 20–50 years (1, 2). In developing countries, approximately 25% of irreversible blindness is caused by uveitis and its complications (3, 4).

Experimental autoimmune uveitis (EAU), an animal model of noninfectious uveitis, shares many common features in clinical and histological aspects with human uveitis (5–7). The most common concept of autoimmune uveitis is that it arises from an impairment of the immune privilege of the eye (8–10). Immune privilege is a complex phenomenon that involves multiple components, starting with the sequestration of antigens behind an efficient blood–retinal barrier (BRB), thereby inhibiting the activation and function of acquired and innate immune cells (11). When the immune privilege is broken in the course of EAU, acquired immune cells, including Th1 and Th17 cells, cross the BRB and initiate retinal destruction, resulting in a distorted structure of the retina (12–14). Recent studies have shown that innate immune cells including macrophages and microglia are involved in antigen presentation during EAU (15). Macrophages are also considered to be important effector cells in EAU *via* the secretion of inflammatory cytokines (16). In addition to blood-borne inflammatory cells, retinal microglia show phagocytic and pathogenic features similar to those exhibited by macrophages. Activated macrophages and retinal microglia release pathogenic factors, such as tumor necrosis factor-α (TNF-α) and inducible nitric oxide synthase (iNOS), resulting in the nitration of cytochrome *c* which is known to cause apoptosis in EAU (17–19). Apoptosis of retinal cells, that cannot be replaced, will cause irreversible damage to the visual system during intraocular inflammation (20). Factors that may affect apoptosis following an inflammatory response include the activation of the aryl hydrocarbon receptor (AhR) (21).

Aryl hydrocarbon receptor is a nuclear receptor belonging to the family of transcription factors of the basic–helix–loop–helix–Per–Arnt–Sim and was initially defined as the receptor for 2,3,7,8-tetrachlorodibenzo-p-dioxin (TCDD), 6-formylindolo [3,2-b] carbazole, and 2-(1H′-indole-3′-carbonyl)-thiazole-4-carboxylic acid methyl ester (22–25). AhR signaling has now been identified to play an essential role in multiple aspects of normal physiology, including vascular development, neurologic function, and vision (26). AhR signaling may also affect inflammation. AhR-deficient mice, for instance, develop more severe symptoms and disease scores in the experimental autoimmune encephalomyelitis model when compared with wild-type mice (27). In the experimental model of age-related macular degeneration, AhR^−/−^ mice exhibited subretinal accumulation of microglia and exacerbated choroidal neovascularization (28, 29). A previous study from our group showed that a decreased AhR expression is associated with disease activity in Behcet’s disease patients (30). A recent study suggested that AhR activation by TCDD was able to suppress EAU by inducing regulatory T cells (31). However, the exact mechanism whereby AhR activation *via* TCDD modulates innate immune response in intraocular inflammation remains unknown and was therefore the subject of this study. We used the EAU model in mice and investigated the role of AhR by using both an AhR knockdown approach and an AhR agonist treatment to detect the effects of AhR on disease manifestations, cytokine response, and signaling pathways.

## Materials and Methods

### Animals

AhR^+/−^ mice in C57BL/6J background, aged 3–12 weeks, were purchased from Jackson Laboratory. Female AhR-deficient mice and wild-type mice used in this study were obtained by breeding AhR-heterozygous (AhR^+/−^) females with AhR^+/−^ males. Mice were housed individually and maintained on a 12-h light/dark cycle with food and water available *ad libitum* under specific pathogen-free conditions in the animal facilities of the Experimental Animal Center of Chongqing Medical University. We extracted genomic DNA from the tail tips and used PCR to determine the AhR status in the offspring. A total of 28 AhR^+/+^ and 28 AhR^−/−^ female mice were divided into four groups: AhR^+/+^ non-immunized group (*n* = 14), AhR^−/−^ non-immunized group (*n* = 14), AhR^+/+^ EAU group (*n* = 14), and AhR^−/−^ EAU group (*n* = 14). Another 42 AhR^+/+^ female mice were divided into three groups: naive mouse group (*n* = 14), vehicle-treated EAU group (*n* = 14), and TCDD-treated EAU group (*n* = 14). All the experimental procedures were carried out conforming to the ARVO statement for the Use of Animals in Ophthalmic and Vision Research. The protocol was approved by the Ethics Committee of the First Affiliated Hospital of Chongqing Medical University (Number: 2016-171). Every effort was made to minimize animal suffering and discomfort.

### Reagents

Human IRBP_651-670_ (LAQGAYRTAVDLESLASQLT) was synthesized and purified by Shanghai Sangon Biological Engineering Technology & Services Ltd. Co. (Shanghai, China). Complete Freund’s adjuvant was obtained from Sigma-Aldrich (St. Louis, MO, USA). Heat-killed *M. tuberculosis* strain H37Ra was obtained from BD Biosciences (NJ, USA), and pertussis toxin was obtained from Sigma-Aldrich (St. Louis, MO, USA). TCDD was purchased from J&K (Beijing, China).

### Induction and Treatment of EAU

To induce EAU, mice were subcutaneously immunized with 500 µg human IRBP_651–670_ in 0.1 ml PBS, emulsified with an equal volume of complete Freund’s adjuvant containing 5 mg/ml *M. tuberculosis* strain H37Ra (1:1 v/v) as previously described (32). Simultaneously, the mouse also received 1 µg *Bordetella pertussis* toxin (Sigma-Aldrich, St. Louis, MO, USA) intraperitoneally. A dose of 1 µg TCDD (dissolved in 0.2 ml olive oil) was administrated intraperitoneally 24 h before the immunization in the TCDD group as previously described (31). Meanwhile, the vehicle group received the equal volume of vehicle (olive oil) in the same way. After immunization, eyes were dilated with tropicamide (0.5%) and examined with a slit-lamp microscope. Clinical severity of ocular inflammation was evaluated by two independent ophthalmologists in a masked fashion and the clinical score of each mouse was graded on a scale of 0–5 in half-point increments, according to five separate criteria described previously (2, 33).

### Hematoxylin and Eosin (H&E) Staining

On day 14 after immunization, six mice per group were euthanized. The eyeballs were enucleated, post-fixed, and immersed in PBS with 10% formaldehyde and 5% glacial acetic acid. The fixed and dehydrated tissues were embedded in paraffin wax, and serial 4–6 µm sections were made through the papillary–optic nerve axis. Subsequently, sections were deparaffinized, hydrated, washed, and stained with H&E. No less than four sections of each eye were assessed histologically according to Caspi’s criteria (6). The severity of EAU was graded on a scale of 0–4 in a masked manner.

### Evans Blue

The microvascular permeability of the BRB was evaluated by Evans blue as previously described (34). Evans blue, an acid dye binding to albumin in the blood, can indicate BRB breakdown. In brief, four mice per group were injected with 100 µl of 2% (wt/vol) Evans blue (Sigma-Aldrich, St. Louis, MO, USA) through the tail veins and euthanized 2 h later. The eyes were enucleated and immediately fixed in fresh 2% (wt/vol) paraformaldehyde for another 2 h. After removing the corneas, lenses, sclera, vitreous, and stripping the retinas from the choroids, the retinas were dissected and washed twice in cold PBS for 15 min. After that, the retinas with the vitreous side facing up were stretched onto clean slides and under cover slips with glycerol. Photographs of the retinal flat mounts were taken with an immunofluorescent microscope (Leica, Germany).

### Western Blotting Analysis

Western blotting analysis was performed using a standard technique as previously described (2). On day 14 after immunization, four mice per group were deeply anesthetized, and retinas were carefully dissected from enucleated eyeballs. Protein was extracted following homogenization with radio immunoprecipitation assay lysis buffer (Beyotime, Shanghai, China) including 1% potease inhibitor (Beyotime, Shanghai, China), and the protein concentration was measured using a bicinchoninic acid assay kit (Beyotime, Shanghai, China). Equal amounts of 50 μg protein were separated by 6–12% SDS-polyacrylamide gel electrophoresis, and the resolved proteins were electroblotted onto polyvinylidene difluoride membranes (Millipore, MA, USA). The membranes were incubated with primary antibodies overnight at 4°C after blocking with 5% nonfat milk. Primary antibodies used included rabbit anti-CD16 (1:20,000, Abcam, UK), rabbit anti-CD206 (1:1,000, Abcam, UK), rabbit anti-iNOS (1:200, Proteintech, Wuhan, China), rabbit anti-arginase-1 (Arg-1) (1:200, Proteintech, Wuhan, China), rabbit anti-occludin (1:200, Proteintech, Wuhan, China), rabbit anti-claudin-5 (1:1,000, Abcam, UK), rabbit anti-zonula occludens-1 (ZO-1) (1:200, Proteintech, Wuhan, China), rabbit anti-cleaved caspase-3 (1:2,000, Abcam, UK), rabbit anti-Bcl-2 (1:500, Wanleibio, Liaoning, China), rabbit anti-Bcl-2 associated X protein (Bax) (1:1,000, Abcam, UK), mouse anti-p65 (1:500, Servicebio, Wuhan, China), rabbit anti-phospho-signal transducers and activators of transcription (STAT)1 (1:500, Cell Signaling Technology, MA, USA), rabbit anti-phospho-STAT3 (1:500, Servicebio, Wuhan, China), and rabbit anti-glyceraldehyde-3-phosphate dehydrogenase (1:2,000, Proteintech, Wuhan, China) was used as a loading control. The membranes were washed in Tris-buffered saline with 0.1% Tween-20 and incubated with HRP-affinipure goat anti-rabbit IgG or HRP-affinipure goat anti-mouse IgG secondary antibodies (1:1,000, Proteintech, Wuhan, China) at room temperature for 1 h. Blots were visualized by using an ECL kit (Advansta, CA, USA) and band densitometry was quantified using Image J software (http://imagej.nih.gov/ij/; provided in public domain by the National Institutes of Health, Bethesda, MD, USA). All experiments were repeated in triplicate.

### Immunofluorescence

Paraffin sections (four mice per group) were fluorescent immunolabeled following a standard indirect technique (primary antibody followed by fluorescent secondary antibody) as previously described (35). Primary antibodies used include goat anti-ionized calcium-binding adaptor molecule 1 (Iba1) (1:200, Abcam, UK). The integrated optic density (IOD) of Iba1 was measured in five randomly selected fields (scale bar, 10 µm) for each section using Image J software and averaged to one number. Three sections of one eye were imaged and averaged.

### TdT-Mediated dUTP Nick End Labeling (TUNEL) Assay

TdT-mediated dUTP nick end labeling staining was performed according to the manufacturer’s instructions and counterstained with DAPI (Roche, UK). TUNEL-positive cells were counted in five randomly selected fields (200×) for each section using Image J software and averaged to one number. Three sections of one eye were imaged and averaged.

### Enzyme-Linked Immunosorbent Assay (ELISA)

After anesthetization, blood sample was collected from each mouse. After centrifugation, the plasma was collected and stored at −80°C. The concentration of plasma TNF-α, interleukin (IL)-6, and IL-1β was measured using ELISA kits for mice (Neobioscience, Guangdong, China).

### Statistical Analysis

All data were expressed as mean ± SD, and statistical calculations were performed using SPSS software 19.0 (IBM, USA). Unpaired Student’s *t*-test was applied to assess significance between two groups. Experimental data for multiple group comparisons were analyzed by one-way ANOVA. Differences in EAU scores were analyzed using the Mann–Whitney *U* test. The differences were considered statistically significant at *p* < 0.05. All statistical figures were made using Prism version 6.0d software (GraphPad, San Diego, CA, USA).

## Results

### More Severe Clinical and Histological Characteristics Were Observed in AhR^−/−^ EAU Mice

**Figure 1:**
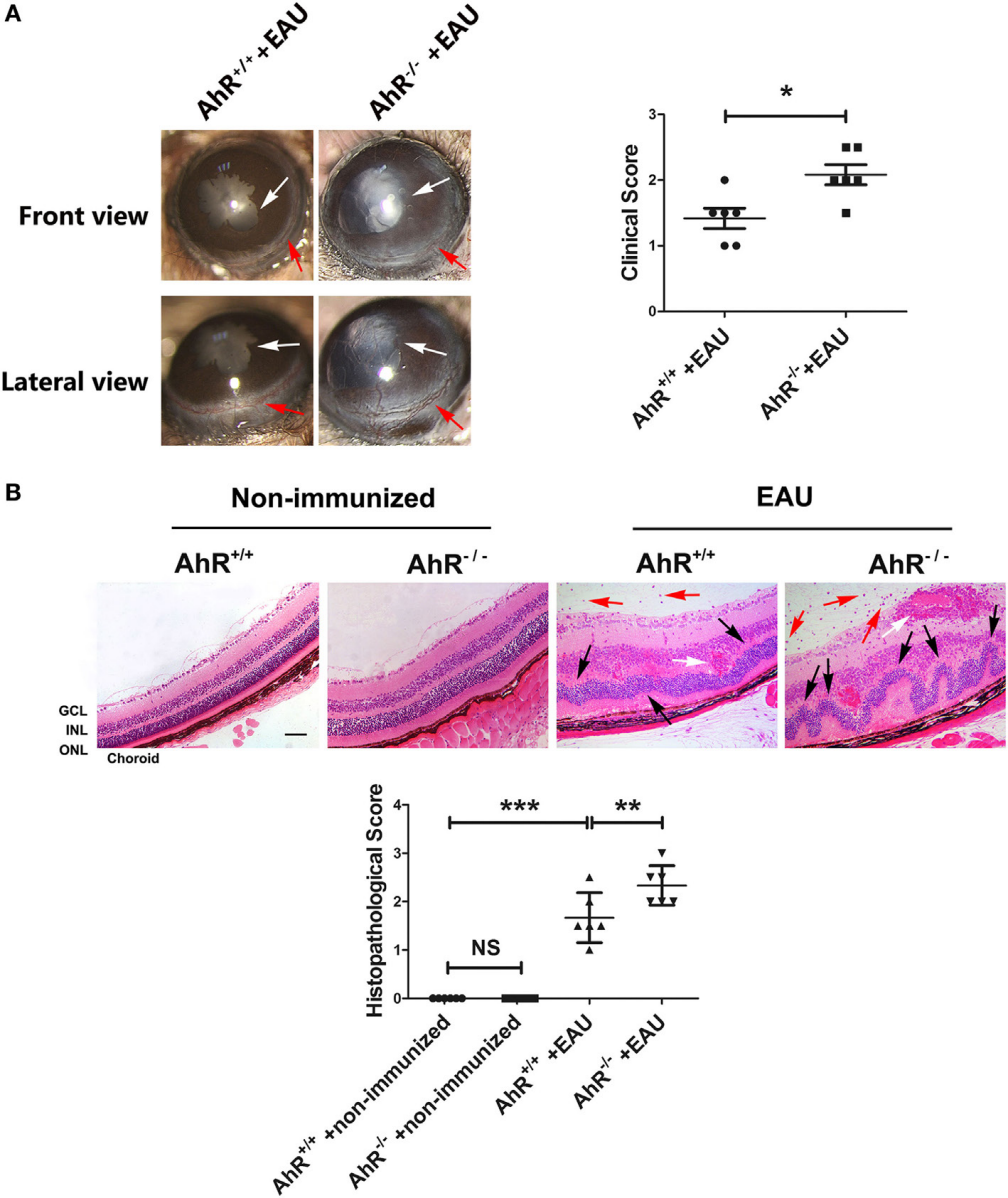
Effect of aryl hydrocarbon receptor (AhR) knockout on clinical and histological characteristics of experimental autoimmune uveitis (EAU). **(A)** Left, representative slit-lamp images of AhR^−/−^ mice and AhR^+/+^ mice after immunization from the frontal and lateral view. White arrow, posterior synechiae. Red arrow, conjunctival hyperemia. Right, quantification of clinical score of AhR^−/−^ mice and AhR^+/+^ mice after EAU (*n* = 6/group; mean ± SD; *p < 0.05; unpaired Student’s *t*-test). **(B)** Upper, representative hematoxylin and eosin images of eye sections in AhR^−/−^ mice and AhR^+/+^ mice after EAU induction or without immunization. Abbreviations: GCL, ganglion cell layer; INL, inner nuclear layer; ONL, outer nuclear layer. Black arrow, retinal fold. Red arrow, vitreous infiltration. White arrow, vasculitis. Scale bar, 30 µm. Lower. Quantification of histopathological score of AhR^−/−^ and AhR^+/+^ EAU mice (*n* = 6/group; mean ± SD; ^NS^*p* > 0.05,^**^ *p* < 0.01; ***p < 0.001; one-way ANOVA).

To determine whether AhR plays a role in the development of EAU, we immunized animals with human IRBP_651–670_ and compared AhR^−/−^ mice with AhR^+/+^ mice. Slit-lamp examination was used to examine the degree of anterior chamber inflammation. The anterior chamber at the 14th day after IRBP immunization showed that the inflammatory signs including conjunctival hyperemia and posterior synechiae were more obvious in AhR^−/−^ mice compared with that in the wild-type animals (Figure 1A).

To evaluate the histological scores, eyes were collected from non-immunized mice or mice immunized with IRBP_651–670_. There was no significant difference between AhR^+/+^ non-immunized mice and AhR^−/−^ non-immunized mice in histological scores. After immunization with IRBP_651–670_, AhR^+/+^ mice showed obvious histopathological signs of uveitis compared with non-immunized mice, as evidenced by the retinal folding, photoreceptor damage, and infiltration of inflammatory cells throughout the retina and choroid. Moreover, AhR^−/−^ EAU mice showed higher histological scores than AhR^+/+^ EAU mice (Figure 1B).

### AhR Knockout Leads to BRB Breakdown in EAU Mice

**Figure 2:**
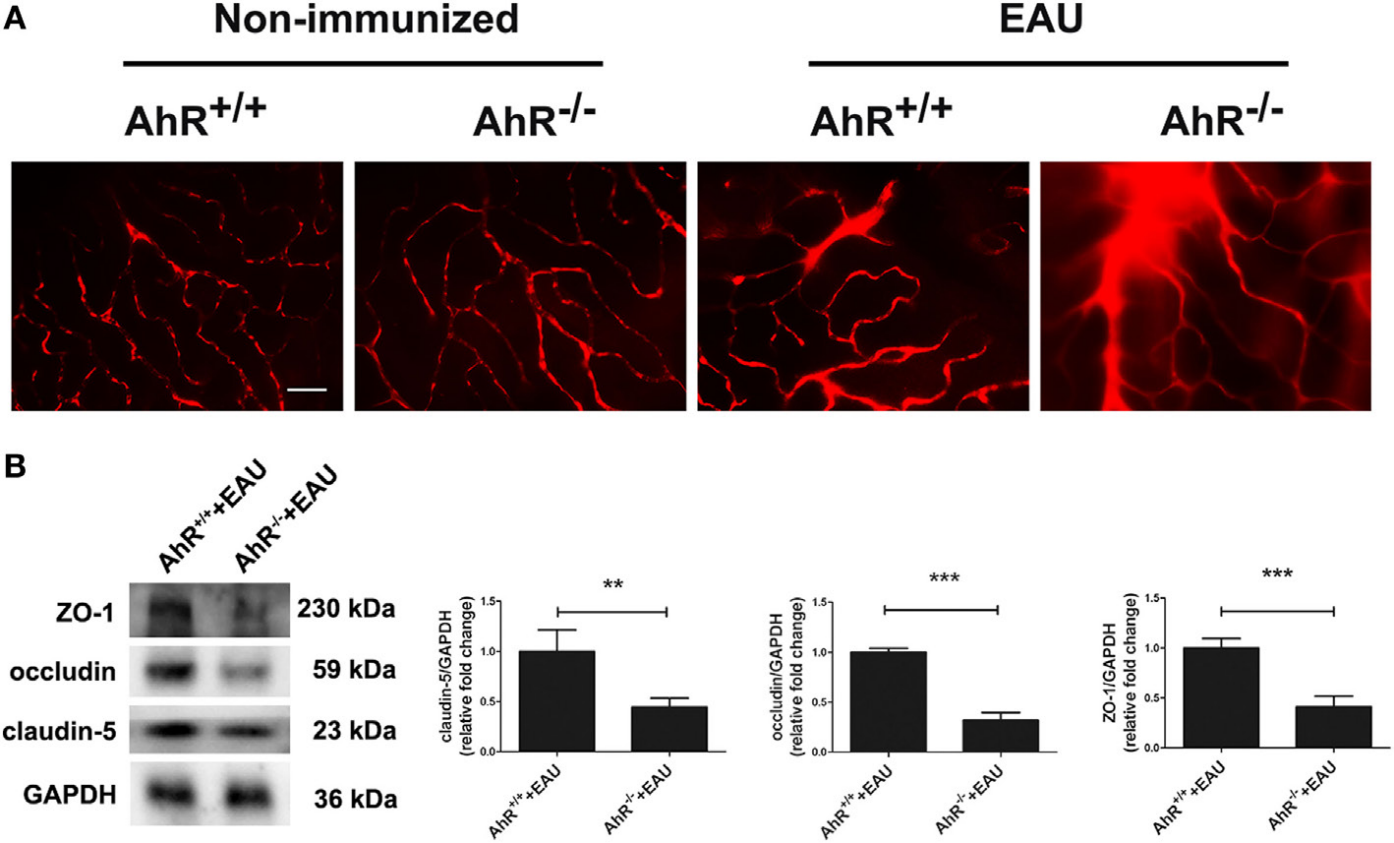
Aryl hydrocarbon receptor (AhR) knockout leads to blood–retinal barrier (BRB) breakdown in experimental autoimmune uveitis (EAU) mice. **(A)** Representative Evans blue images of retinas in AhR^−/−^ and AhR^+/+^ non-immunized or EAU mice. Scale bar, 30 µm (*n* = 4/group). **(B)** Left, representative Western blotting images of claudin-5, occludin and zonula occludens-1 (ZO-1) in retinas of AhR^−/−^ and AhR^+/+^ EAU mice. Right, quantification of relative fold change of claudin-5, occluding, and ZO-1 expressions (*n* = 4/group; mean ± SD; **p < 0.01; ***p < 0.001; unpaired Student’s *t*-test).

To investigate whether AhR knockout caused BRB breakdown, the BRB integrity was examined using Evans blue dye and the expression of the tight junction proteins ZO-1, claudin-5, and occludin was measured. No Evans blue leakage was observed in AhR^+/+^ and AhR^−/−^ non-immunized mice. Diffuse Evans blue leakage from the retinal vasculature was commonly investigated in AhR^+/+^ EAU mice and was stronger in AhR^−/−^ EAU mice (Figure 2A). Furthermore, the retinal expression of tight junction proteins ZO-1, claudin-5, and occludin was much lower in AhR^−/−^ EAU mice than in AhR^+/+^ EAU mice (Figure 2B).

### AhR Knockout Exacerbated Apoptosis in EAU Mice

**Figure 3:**
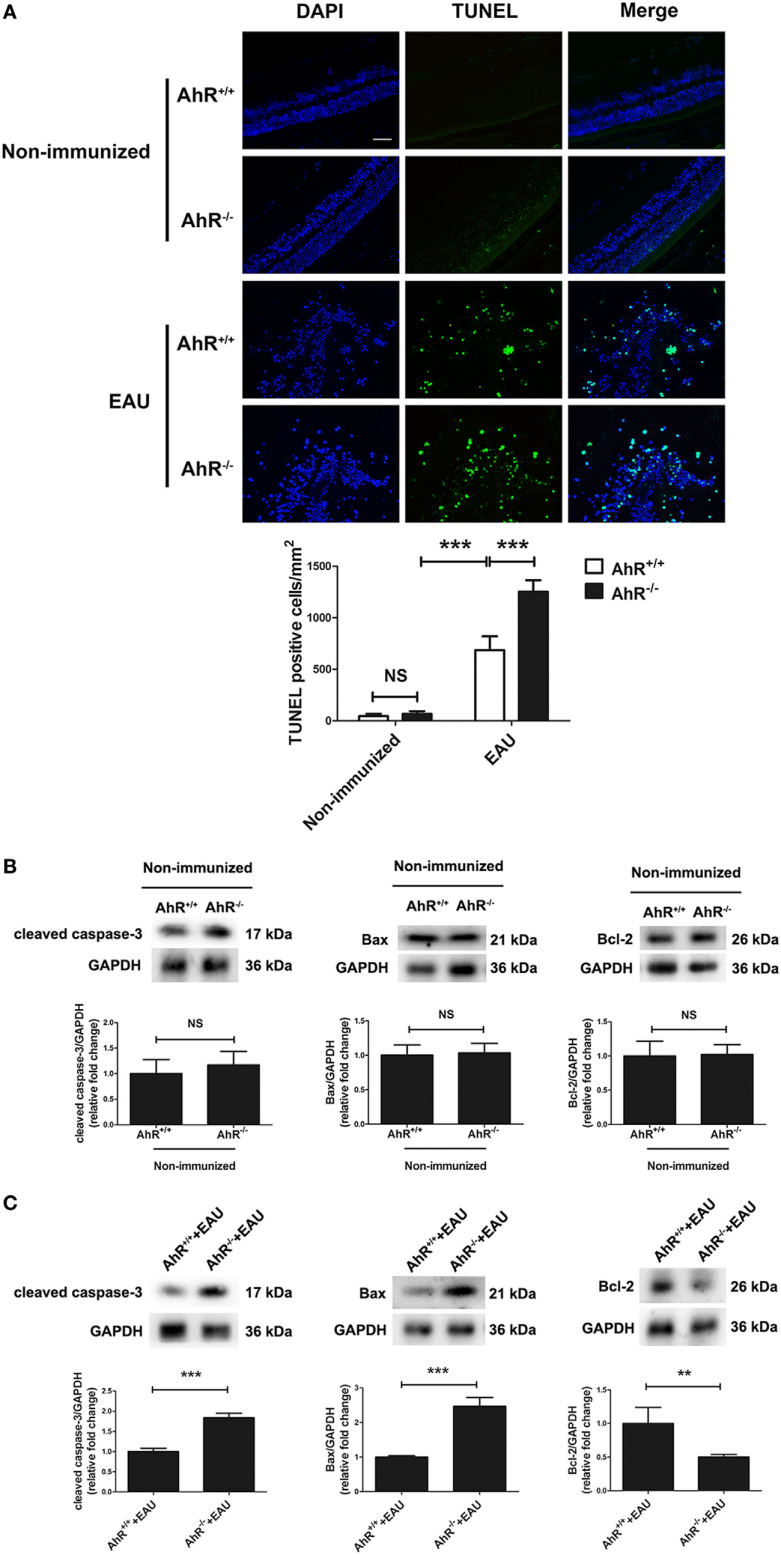
Aryl hydrocarbon receptor (AhR) knockout increased apoptosis in experimental autoimmune uveitis (EAU) mice. **(A)** Upper, representative TdT-mediated dUTP nick end labeling (TUNEL) Images of eye sections in AhR^−/−^ and AhR^+/+^ non-immunized or EAU mice. Scale bar, 30 µm. Lower, quantification of TUNEL-positive cells (*n* = 4/group; mean ± SD; ^NS^*p* > 0.05, ***p < 0.001; one-way ANOVA). **(B)** Upper, representative Western blotting images of cleaved caspase-3, Bcl-2 associated X protein (Bax), and Bcl-2 in retinas of AhR^−/−^ and AhR^+/+^ non-immunized mice. Lower, quantifications of relative fold change of cleaved caspase-3, Bax, and Bcl-2 expressions (*n* = 4/group; mean ± SD;^NS^ *p* > 0.05; unpaired Student’s *t*-test). **(C)** Upper, representative Western blotting images of cleaved caspase-3, Bax, and Bcl-2 in retinas of AhR^−/−^ and AhR^+/+^ EAU mice. Lower, quantifications of relative fold change of cleaved caspase-3, Bax, and Bcl-2 expressions (*n* = 4/group; mean ± SD; **p < 0.01; ***p < 0.001; unpaired Student’s *t*-test).

To evaluate whether the presence of AhR affects retinal cell apoptosis in EAU mice, we performed TUNEL staining of retinal sections. The results indicate that AhR^+/+^ and AhR^−/−^ non-immunized mice showed little TUNEL-positive cells, whereas AhR^+/+^ EAU mice exhibited increased TUNEL-positive cells compared with AhR^+/+^ non-immunized mice. However, more TUNEL-positive cells were found in AhR^−/−^ EAU mice than in AhR^+/+^ EAU mice (Figure 3A). We further investigated the mechanism of cell death by measuring the protein levels of cleaved caspase-3, Bax, and Bcl2 in non-immunized mice or EAU mice. Caspase-3 is known to be an important molecule in the cellular apoptotic cascade (36). Bax and Bcl-2, two members of the Bcl-2 family, exhibit pro-apoptotic and anti-apoptotic properties, respectively, interacting with caspase signals. AhR^−/−^ non-immunized mice showed no significant difference in the expressions of cleaved caspase-3, Bax, and Bcl-2 when compared with that in AhR^+/+^ non-immunized mice (Figure 3B). However, the expressions of both cleaved caspase-3 and Bax was significantly increased in AhR^−/−^ EAU mice compared with AhR^+/+^ EAU mice, whereas the Bcl-2 protein level was markedly decreased in AhR^−/−^ EAU mice when compared with AhR^+/+^ EAU mice (Figure 3C).

### Deletion of AhR in Mice Resulted in Increased Recruitment of Macrophages/Microglia

**Figure 4:**
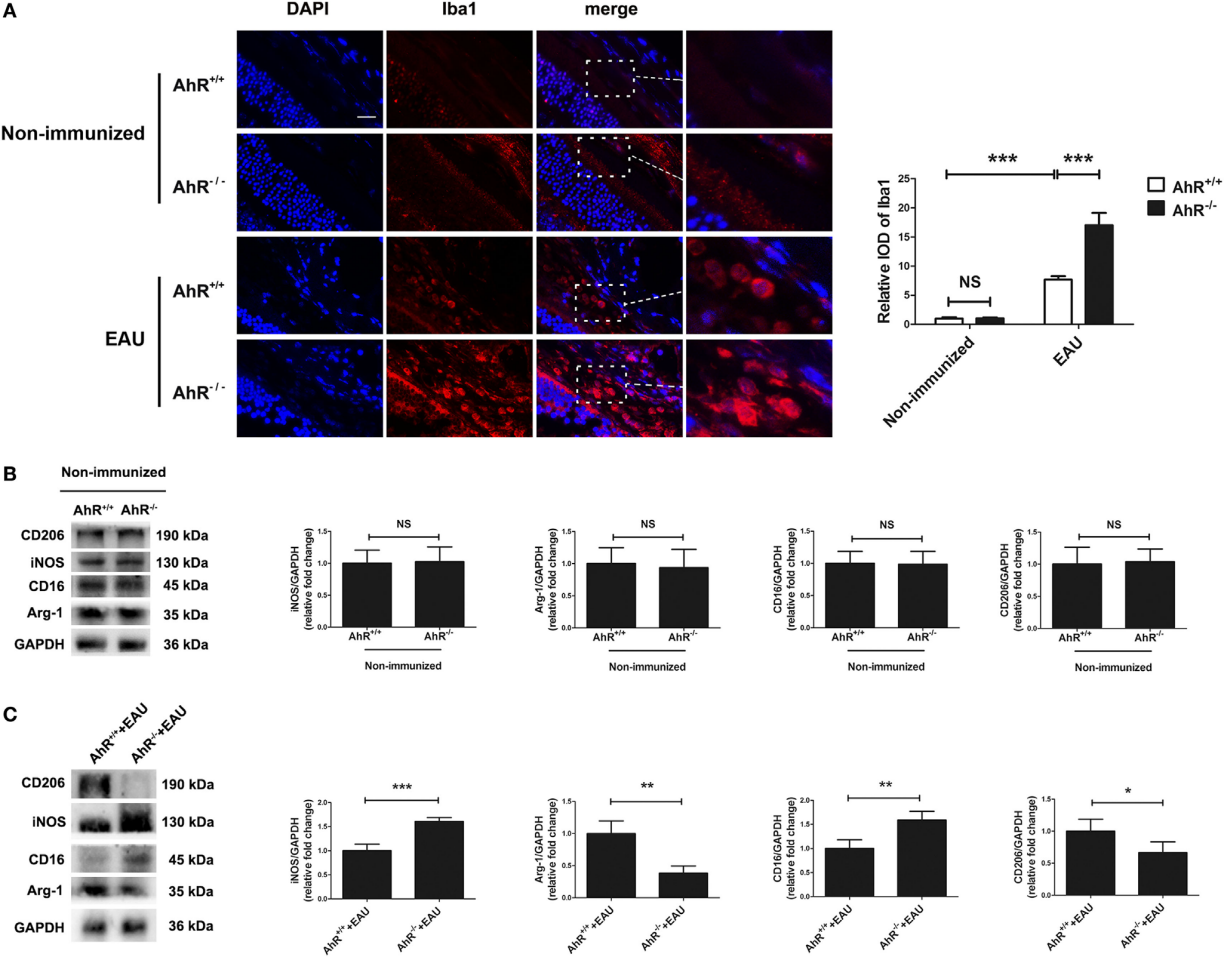
Effect of aryl hydrocarbon receptor (AhR) deletion on the recruitment of macrophages/microglia in experimental autoimmune uveitis (EAU) mice. **(A)** Left, representative immunofluorescent images of ionized calcium-binding adaptor molecule 1 (Iba1) (a marker of macrophage/microglia) and DAPI (nuclei) in retinas of AhR^−/−^ and AhR^+/+^ EAU or non-immunized mice. Scale bar, 10 µm. Right, quantification of relative integrated optic density (IOD) of Iba1 (*n* = 4/group; mean ± SD;^NS^ *p* > 0.05, ***p < 0.001; one-way ANOVA). **(B)** Left, representative Western blotting images of inducible nitric oxide synthase (iNOS), arginase-1 (Arg-1), CD16 and CD206 in retinas of AhR^−/−^ and AhR^+/+^ non-immunized mice. Right, quantifications of relative fold change of iNOS, Arg-1, CD16, and CD206 expressions (*n* = 4/group; mean ± SD;^NS^ *p* > 0.05; unpaired Student’s *t*-test). **(C)** Left, representative Western blotting images of iNOS, Arg-1, CD16, and CD206 in retinas of AhR^−/−^ and AhR^+/+^ EAU mice. Right, quantifications of relative fold change of iNOS, Arg-1, CD16, and CD206 expressions (*n* = 4/group; mean ± SD;^*^ *p* < 0.05, **p < 0.01; ***p < 0.001; unpaired Student’s *t*-test).

Macrophages are considered to be an important effector in EAU *via* the production of inflammatory cytokines. Microglia, a glial cell of the central nervous system, belongs to the hematopoietic system and is involved in inflammatory and immune responses (37). It is also a prominent participant in retinal responses to injury and plays a role in tissue repair (38). Activation of the classical macrophage/microglia (M1 type) caused by IFN-γ induces the production of IL-1β, TNF-α, and NO from iNOS and functions as a cytotoxic phenotype. The other subset, which is known as the M2 type macrophage/microglia, is activated by IL-4 and IL-13, and is involved in tissue repair (39, 40). In EAU, macrophages and microglia can augment the inflammatory reaction and increase the damage to photoreceptors and retina (41). To examine whether the deletion of AhR could affect the activity and recruitment of macrophages/microglia, we performed immunofluorescence of Iba-1 in the retinas of AhR^+/+^ and AhR^−/−^ non-immunized mice. The IOD of Iba1 in the retinas showed no significant difference between AhR^−/−^ and AhR^+/+^ non-immunized mice. However, AhR^+/+^ EAU mice showed increased IOD of Iba1 when compared with AhR^+/+^ non-immunized mice. Moreover, the IOD of Iba1 was higher in AhR^−/−^ EAU mice than in AhR^+/+^ EAU mice (Figure 4A). To study whether AhR is related to macrophage/microglia polarization, we tested the expressions of markers of the M1 subset (iNOS, CD16) and the M2 subset (Arg-1, CD206) by Western blotting analysis. The results showed no significant difference in the expressions of iNOS, CD16, Arg-1, and CD206 between the AhR^+/+^ and AhR^−/−^ non-immunized groups (Figure 4B). However, the expressions of iNOS and CD16 was much higher in AhR^−/−^ EAU mice than in AhR^+/+^ EAU mice, whereas the expressions of Arg-1 and CD206 in AhR^−/−^ EAU mice was downregulated compared with AhR^+/+^ EAU mice (Figure 4C).

### Activation of AhR *via* TCDD Treatment Ameliorated the Clinical Signs and Histological Manifestations in EAU

**Figure 5:**
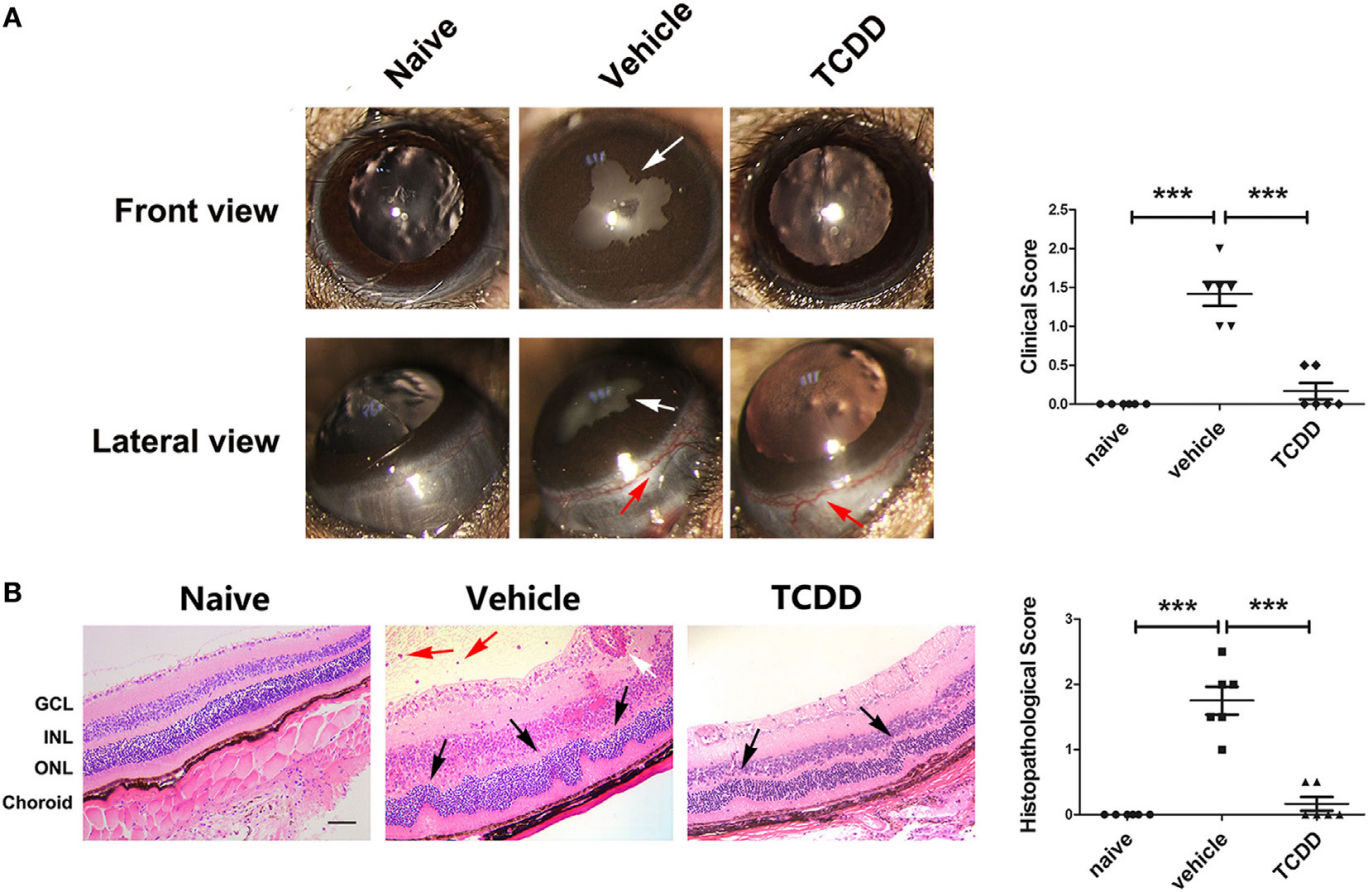
TCDD treatment ameliorated the clinical and histological manifestations in experimental autoimmune uveitis (EAU) mice. **(A)** Left, representative slit-lamp images of eye sections in naive, vehicle, and TCDD-treated mice. White arrow, posterior synechiae. Red arrow, conjunctival hyperemia. Right, quantification of clinical score (*n* = 6/group; mean ± SD; ***p < 0.001; one-way ANOVA). **(B)** Left, representative hematoxylin and eosin images of eye sections in naive, vehicle and TCDD-treated mice. Black arrow, retinal fold. Red arrow, vitreous infiltration. White arrow, vasculitis. Scale bar, 30 µm. Right, quantification of histopathological score (*n* = 6/group; mean ± SD; ***p < 0.001; one-way ANOVA).

To investigate the therapeutic effect of TCDD on the EAU model, AhR^+/+^ EAU mice were treated with vehicle or TCDD. The expression of AhR in nuclei was decreased in vehicle-treated EAU mice compared with naive mice, whereas TCDD-treated mice showed increased nuclear AhR expression compared with vehicle controls, demonstrating that TCDD increases the nuclear expression of AhR (Figure S1 in Supplementary Material). Slit-lamp examination was used to examine the disease severity in naive mice, vehicle-treated EAU mice, and TCDD-treated EAU mice. Vehicle-treated mice exhibited obvious clinical signs of EAU after immunization, which progressed gradually until day 14. No obvious inflammatory manifestations were seen in the TCDD-treated mice (Figure 5A).

To further validate the therapeutic effects of TCDD, a histological assessment was performed on day 14 post-immunization with IRBP_651–670_. More severe retinal folds and more inflammatory cells were seen in vehicle-treated EAU mice than in TCDD-treated EAU mice (Figure 5B). This confirms that activation of the AhR *via* low dose TCDD treatment significantly reduced the histological damage.

### Activation of AhR *via* TCDD Treatment Reduced Vascular Leakage and Breakdown of the BRB in EAU

**Figure 6:**
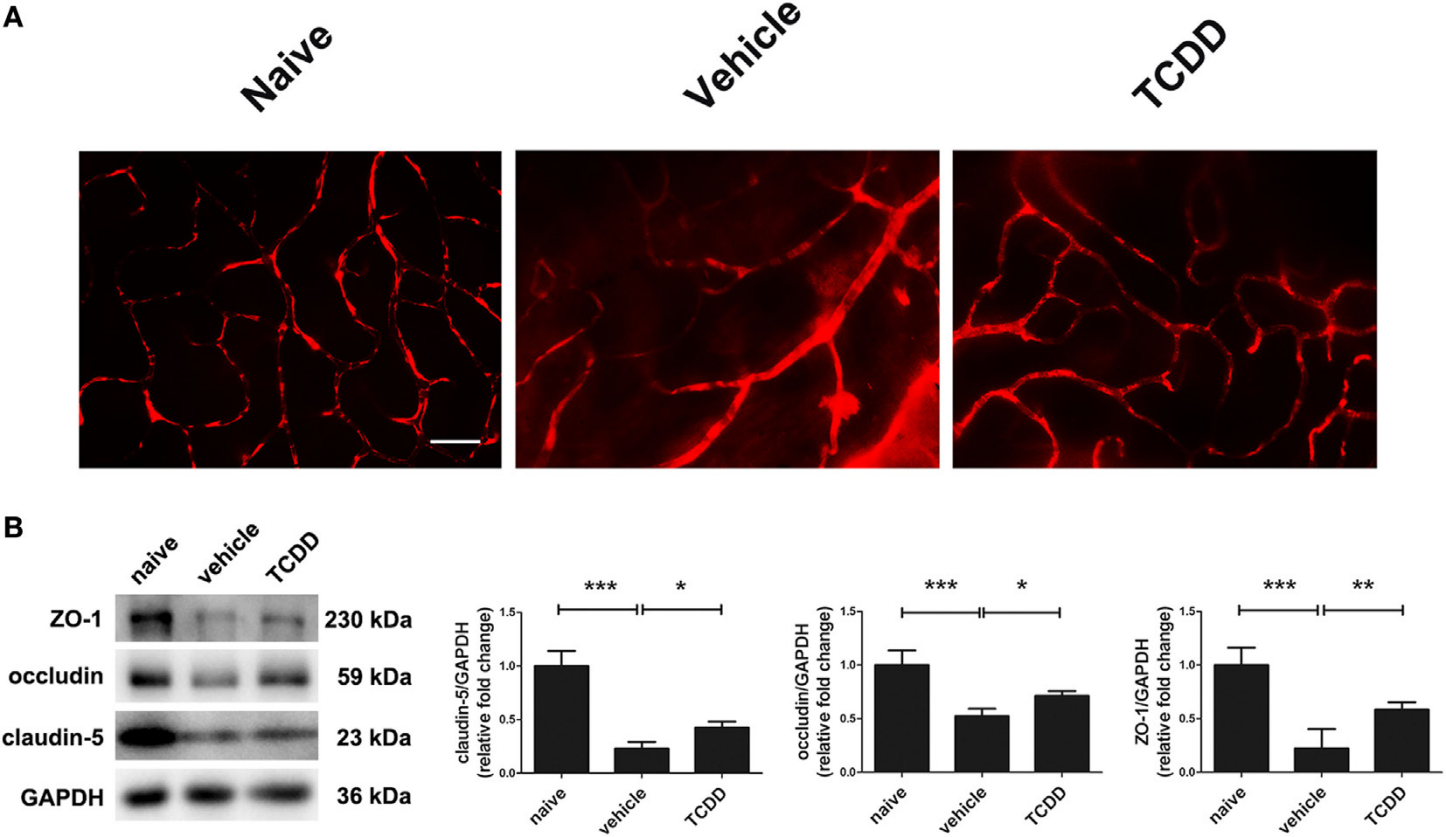
TCDD treatment alleviated blood–retinal barrier breakdown. **(A)** Representative Evans blue images of retinas in naive, vehicle, and TCDD-treated mice. Scale bar, 30 µm. **(B)** Left, representative western blotting images of claudin-5, occluding, and zonula occludens-1 (ZO-1) in retinas naive, vehicle, and TCDD-treated mice. Right, quantifications of relative fold change of claudin-5, occludin, and ZO-1 expressions (*n* = 4/group; mean ± SD; *p < 0.05; **p < 0.01; ***p < 0.001; one-way ANOVA).

To further evaluate the immunomodulatory effect of low dose TCDD on retinas, the BRB integrity was examined using Evans blue dye, and the expression of the tight junction proteins ZO-1, claudin-5, and occludin was measured in naive mice, vehicle-treated EAU mice, and TCDD-treated EAU mice. Massive leakage around the retinal vessels was observed in vehicle-treated mice. On the contrary, only a slight leakage was observed in the low dose TCDD-treated mice (Figure 6A). In addition, the expression of tight junction proteins ZO-1, claudin-5, and occludin in the retinas was much lower in vehicle-treated mice than in TCDD-treated mice (Figure 6B).

### Activation of AhR *via* TCDD Treatment Decreased the Number of Apoptotic Cells and Modulated Cell Death Signals in the Retinas of EAU Mice

**Figure 7:**
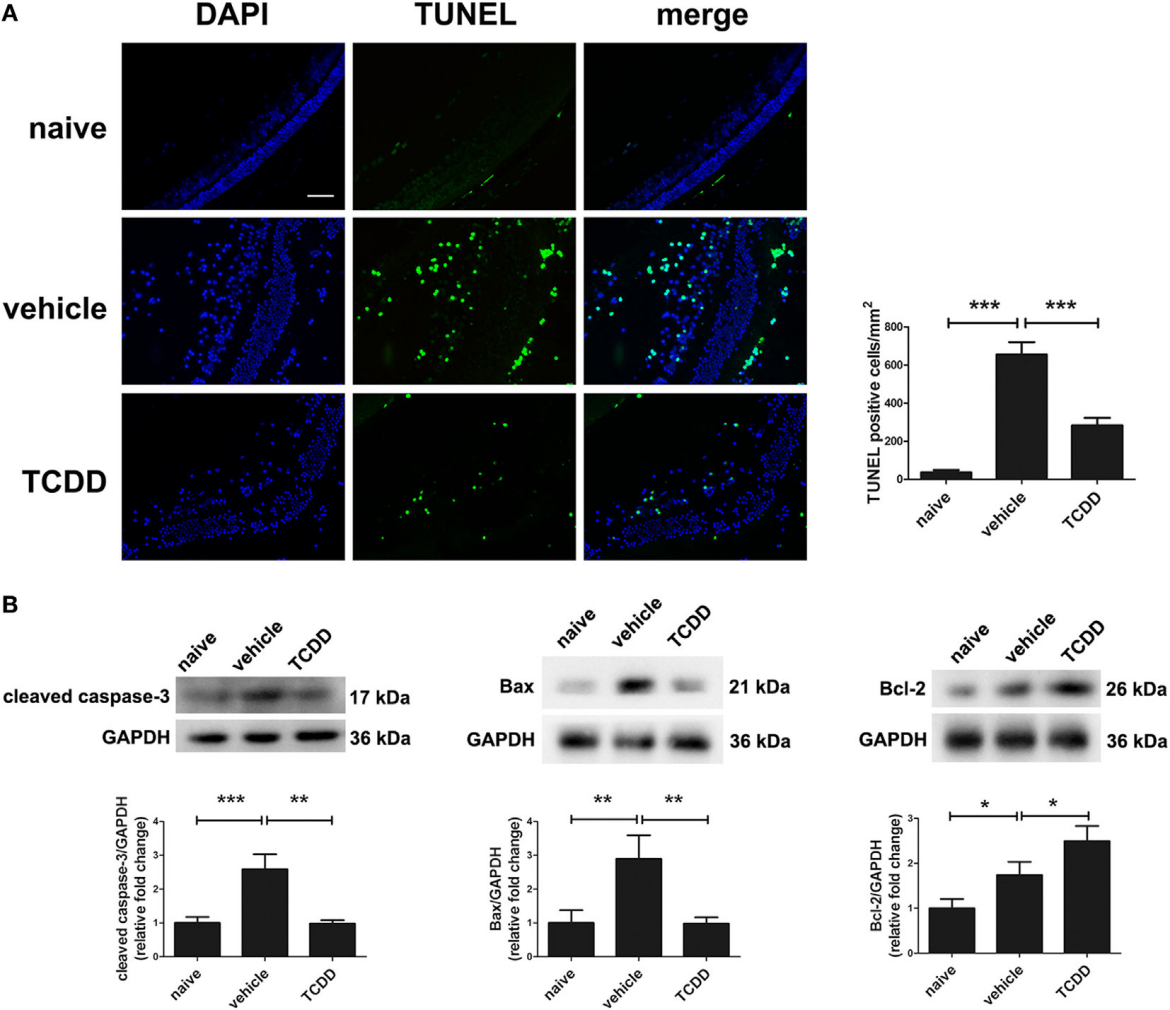
TCDD treatment decreased apoptotic cell death in the retinas of experimental autoimmune uveitis mice. **(A)** Left, representative TdT-mediated dUTP nick end labeling (TUNEL) images of eye sections in naive, vehicle, and TCDD-treated mice. Scale bar, 30 µm. Right, quantification of TUNEL-positive cells (*n* = 4/group; mean ± SD;^***^ *p* < 0.001; one-way ANOVA). **(B)** Upper, representative western blotting images of cleaved caspase-3, Bcl-2 associated X protein (Bax), and Bcl-2 in retinas of naive, vehicle, and TCDD-treated mice. Lower, quantifications of relative fold change of cleaved caspase-3, Bax, and Bcl-2 expressions (*n* = 4/group; mean ± SD;^*^ *p* < 0.05; **p < 0.01; ***p < 0.001; one-way ANOVA).

TdT-mediated dUTP nick end labeling staining was applied on retinal sections to investigate whether TCDD affects apoptosis in mice undergoing EAU. No TUNEL-positive cells were observed in the naive mice. Mice treated with TCDD had a significantly lower number of TUNEL-positive cells in their retinas when compared with vehicle-treated mice (Figure 7A). We further investigated the mechanism of cell death and the immunomodulatory effects of TCDD in the retina by measuring the protein levels of cleaved caspase-3, Bax, and Bcl2 on day 14 after IRBP immunization. As shown in Figure 7B, the expression of cleaved caspase-3 and Bax was significantly decreased after TCDD treatment. On the other hand, the Bcl-2 protein level was significantly increased in the TCDD-treated group when compared with the vehicle-treated group.

### Activation of AhR *via* TCDD Treatment Shifted the Polarization of M1 to M2 Macrophages/Microglia

**Figure 8:**
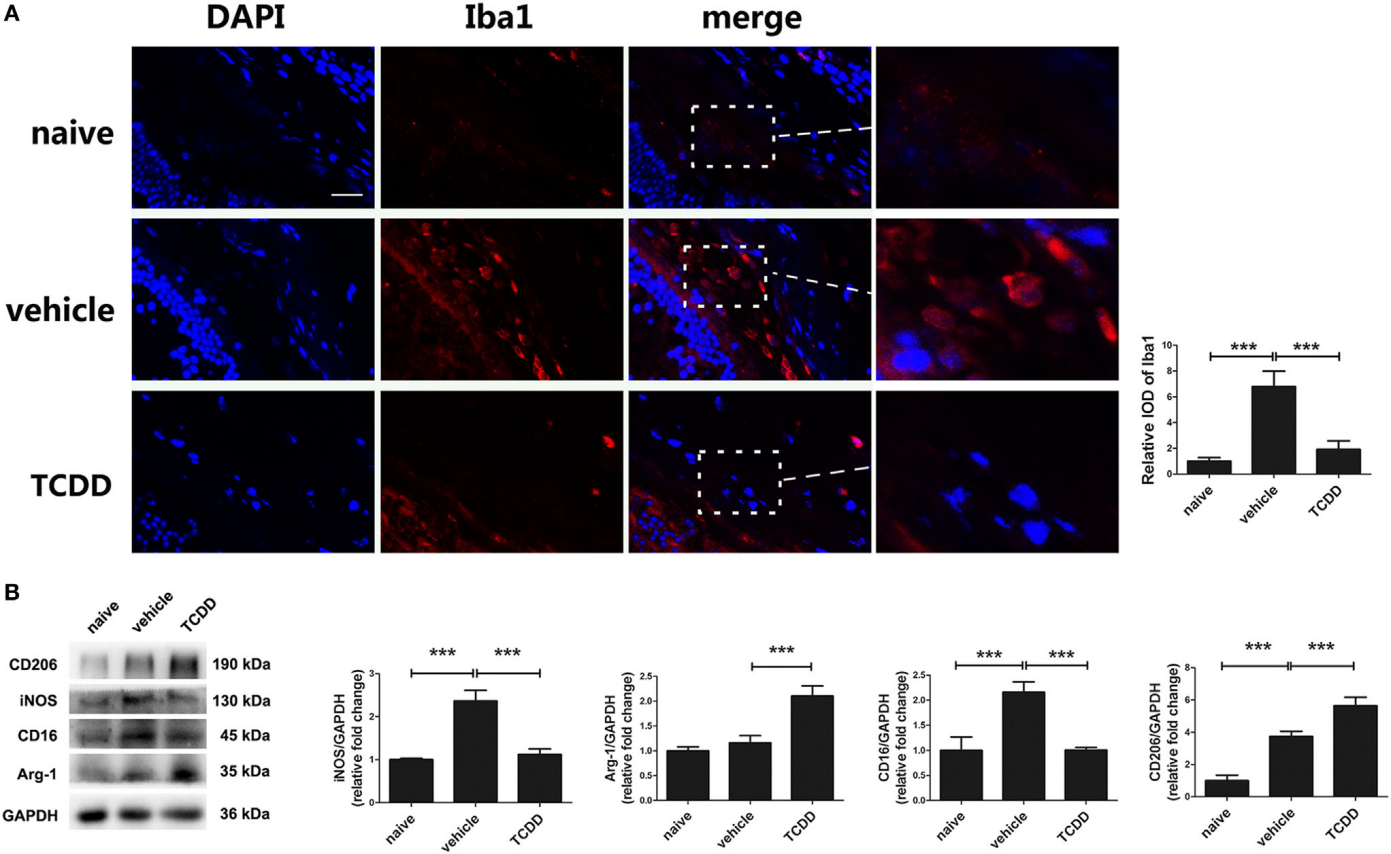
TCDD treatment shifted the polarization of macrophages/microglia from M1 to M2 in experimental autoimmune uveitis mice. **(A)** Left, representative immunofluorescent images of ionized calcium-binding adaptor molecule 1 (Iba1) and DAPI in retinas of naive, vehicle, and TCDD-treated mice. Scale bar, 10 µm. Right, quantification of relative integrated optic density (IOD) of Iba1 (*n* = 4/group; mean ± SD;^***^ *p* < 0.001; one-way ANOVA). **(B)** Left, representative western blotting images of inducible nitric oxide synthase (iNOS), arginase-1 (Arg-1), CD16, and CD206 in retinas of naive, vehicle, and TCDD-treated mice. Right, quantifications of relative fold change of iNOS, Arg-1, CD16, and CD206 expressions (*n* = 4/group; mean ± SD;^***^ *p* < 0.001; one-way ANOVA).

To detect whether the activity of pathogenic macrophages/microglia would be repressed by TCDD treatment, we performed immunofluorescent staining of Iba-1 in retinas of EAU mice. Immunofluorescence labeling with Iba-1 showed that the IOD of Iba1 in the retinas of TCDD-treated mice was significantly reduced compared with vehicle-treated mice (Figure 8A). Since a shift in the polarization of macrophage/microglia subtypes may affect the severity of EAU, we used Western blotting analysis of markers of M1 (iNOS, CD16) and M2 (Arg-1, CD206) subtypes to detect whether TCDD could regulate the polarization of macrophages/microglia. The results showed that the protein level of iNOS and CD16 was significant decreased, while that of Arg-1 and CD206 was increased in the TCDD-treated mice in comparison with vehicle-treated mice. However, the protein level of Arg-1 was not significantly different between naive mice and vehicle mice (Figure 8B).

### Activation of AhR *via* TCDD Treatment Downregulated Pro-Inflammatory Cytokines

**Figure 9:**
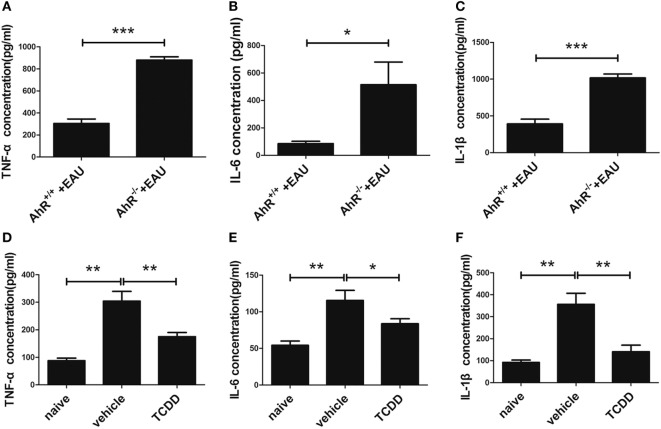
TCDD treatment downregulated pro-inflammatory cytokines in experimental autoimmune uveitis (EAU) mice. **(A–C)** Enzyme-linked immunosorbent assay (ELISA) for testing the protein level of tumor necrosis factor-α (TNF-α), interleukin (IL)-6, and IL-1β in the plasma of AhR^−/−^ and AhR^+/+^ EAU mice (*n* = 4/group; mean ± SD;^*^ *p* < 0.05; ***p < 0.001; unpaired Student’s *t*-test). **(D–F)** ELISA for detecting the protein level of TNF-α, IL-6, and IL-1β in the plasma of naive, vehicle, and TCDD-treated mice (*n* = 4/group; mean ± SD;^*^ *p* < 0.05, **p < 0.01; one-way ANOVA).

The classically activated macrophages/microglia induce the production of TNF-α and NO from iNOS and function as a cytotoxic phenotype (19). To identify which vital cytokines are involved in the immunomodulatory effect of TCDD on EAU pathogenesis, the protein levels of pro-inflammatory mediators (TNF-α, IL-6, and IL-1β) were measured by ELISA. Compared with AhR^+/+^ EAU mice, AhR^−/−^ EAU mice showed higher levels of TNF-α, IL-6, and IL-1β in plasma (Figures 9A–C). The expression of TNF-α, IL-6, and IL-1β in plasma was decreased in the TCDD-treated mice in contrast with the vehicle-treated mice (Figures 9D–F), which was in agreement with the decreased M1 subset levels described above (Figure 8B).

### Activation of AhR *via* TCDD Treatment Inhibited NF-κB and STAT Signaling Pathways

**Figure 10:**
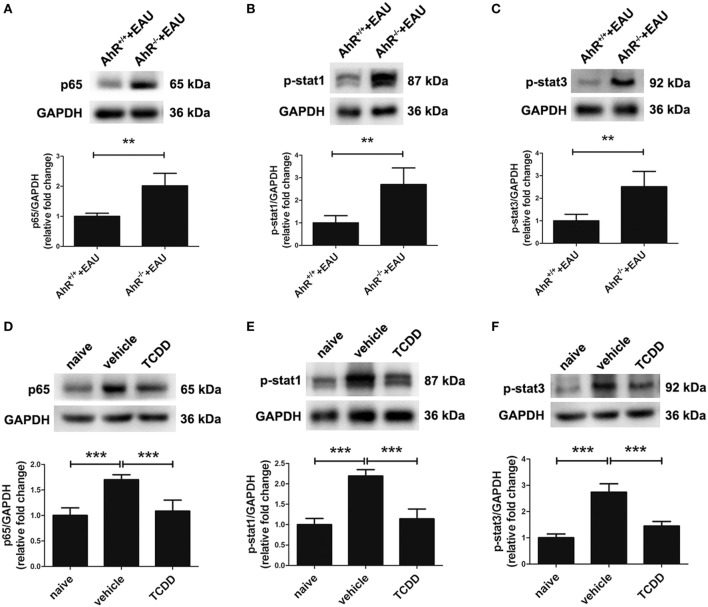
Aryl hydrocarbon receptor (AhR) activation inhibited nuclear factor (NF-κB) and signal transducers and activators of transcription (STAT) signaling pathways in experimental autoimmune uveitis (EAU) mice. **(A–C)** Representative western blotting images of NF-κB p65, p-stat1, and p-stat3 in retinas of AhR^−/−^ and AhR^+/+^ EAU mice and their quantifications (*n* = 4/group; mean ± SD;^**^ *p* < 0.01; unpaired Student’s *t*-test). **(D–F)** Representative Western blotting images of NF-κB p65, p-stat1, and p-stat3 in retinas of naive, vehicle, and TCDD-treated mice and their quantifications (*n* = 4/group; mean ± SD; ***p < 0.001; one-way ANOVA).

To elucidate whether the NF-κB, STAT cell signaling pathways involved in the regulation of pro-inflammatory cytokines by TCDD treatment, the expression levels of NF-κB p65, STAT1, and STAT3 phosphorylated forms were tested with Western blot assays. Our results showed that the protein expression of p65, p-STAT1, and p-STAT3 was elevated in the retinas of AhR^−/−^ EAU mice compared with AhR^+/+^ EAU mice and this could be blocked by TCDD treatment (Figures 10A–C). Moreover, the expression of p65, p-STAT1, and p-STAT3 was downregulated in TCDD-treated mice when compared with vehicle-treated mice (Figures 10D–F).

## Discussion

This study shows that AhR, a ligand-activated nuclear receptor, has a profound effect on the development of autoimmune uveitis in mice. Evidence for this statement comes from two approaches we used. First of all, AhR-knockout mice exhibited more severe clinical and histological manifestations of intraocular inflammation when compared with wild-type mice after EAU induction with a uveitogenic peptide of the retinal protein IRBP. In the second place, we showed that a low dose of the AhR ligand, TCDD markedly suppressed the manifestations of EAU. This treatment was associated with a reduced breakdown of the BRB, a shift in the polarization of macrophages/microglia from the M1 to the M2 subtype, a decrease in the number of apoptotic cells, and a decreased expression of pro-inflammatory cytokines in the blood. The effects mentioned above were probably due to an effect of AhR activation by TCDD on NF-κB, STAT1, and STAT3 cell signaling pathways. These findings in mice are in agreement with our previous observations, in which we found that AhR expression was associated with disease activity in uveitis patients (42).

TCDD, a member of the dioxin family and the most relevant physiological ligand of AhR, is considered as one of the most toxic pollutants on earth and its pathogenicity for humans is a topic of ongoing debate in the field of toxicology (43). Besides its toxicological involvement, attention has recently shifted toward a role of AhR in the immune response. AhR activation by TCDD has for instance been shown to boost T_reg_ cell differentiation and to interfere with the development of Th1 and Th17 cells (27, 31).

Our data are in agreement with an earlier study showing that activation of AhR with TCDD could inhibit the development of EAU (31). We expanded these observations and provided further details concerning the inhibition of uveitis by AhR activation. Our study is the first to analyze the role of AhR in BRB breakdown which is a prominent feature of uveitis (44, 45). Immunization with IRBP in the presence of appropriate adjuvant leads to priming of autoreactive T cells in peripheral lymphoid organs and polarization into activated T_H_ cells which then home to the eye, where they induce BRB breakdown and subsequent massive recruitment of diverse immune cells responsible for tissue damage (46). BRB integrity is maintained by tight junctions which are redistributed and destroyed in the presence of inflammatory cytokines and chemokines in EAU (45). Our study showed that AhR knockout increased vascular leakage and deteriorated the breakdown of BRB indicated by increased Evans blue leakage and the downregulation of the tight junction proteins occludin, claudin-5, and ZO-1. On the contrary, AhR activation *via* TCDD preserved BRB integrity, thereby reducing vascular leakage. BRB breakdown was further proven to be associated with apoptosis of retinal cells (47). In our study, AhR knockout increases cell apoptosis in mice of EAU, while AhR activation with TCDD inhibits apoptosis. Altogether, these findings indicate that AhR preserves BRB integrity in EAU, an effect that may be associated with an anti-apoptotic mechanism.

When the BRB is destroyed, macrophages are recruited to attack intraocular tissues in EAU and increase the damage of photoreceptors and retina (7, 41). Macrophages play dynamic roles in the pathogenesis of autoimmune-mediated inflammatory diseases and they are the main effector cells for tissue damage in EAU (48). Retinal microglia, like their counterpart in the brain, are derived from a myeloid lineage and their progenitors initially enter the nervous system during embryonic and fetal periods (49). Microglia are also prominent participants in retinal responses to injury and play an important role in damage repair (38, 50). As previously reported, microglia migrate to the photoreceptor cell layer and generate TNF-α and peroxynitrite early in the course of EAU, before macrophage infiltration (51). In this study, we found that deletion of AhR in mice was associated with an accumulated recruitment of macrophages/microglia in the retina during EAU. On the other hand, activation of AhR *via* TCDD reduced the number of macrophages/microglia in the retinas of EAU mice. These findings, concerning the role of AhR activation by TCDD on the function of macrophage/microglia, are in agreement with those reported earlier in several other autoimmune models in mice (28, 29, 52, 53). In this study, we also provide evidence for a role of AhR in shifting the polarization of M1 to M2 type of macrophages/microglia, indicating that the protective role of AhR on the BRB might be dependent on regulating macrophage/microglia polarization which is an important process during retinal diseases (2, 54, 55).

As described above, the pro-inflammatory cytokines such as TNF-α, IL-6, and IL-1β contribute to the recruitment and activation of M1 macrophages, leading to augmented ocular inflammation (7). We observed that TCDD treatment suppressed the levels of pro-inflammatory cytokines TNF-α, IL-6, and IL-1β. This anti-inflammatory effect of TCDD may be due to its modulation of macrophages/microglia, as the activated M1 subset accelerates inflammation and causes tissue damage by increasing the secretion of multiple pro-inflammatory cytokines such as TNF-α, IL-6, and the release of reactive oxygen species, resulting in the nitration of cytochrome *c* which is known to cause apoptosis (7, 17, 18). These findings indicate that the anti-inflammatory effect of AhR may contribute to its protection on BRB as well as the control of apoptosis.

During inflammation, a cascade of intracellular signaling pathways will be initiated and ultimately lead to the activation of macrophage/microglia and release of pro-inflammatory cytokines. The major intracellular signaling events include the STAT and NF-κB pathways (2). In this study, we report that AhR inhibits the activity of NF-κB P65, STAT1, and STAT3, indicating that the suppression of ocular inflammation might result, at least, partly from NF-κB and STAT pathway inhibition.

Aryl hydrocarbon receptor plays an important role in regulating systematic and local immunity. On the one hand, the loss of AHR may lead to a systemic inflammatory response as AhR null mice were highly susceptible to autoimmunity diseases, such as dextran sodium sulfate-induced colitis and experimental autoimmune encephalomyelitis (52, 56). However, the activation of AhR is related to differentiation of regulatory T cells and suppression of autoimmune disease (57–60). On the other hand, AhR deficiency also loses the ability to regulate ocular inflammation (29, 61). In our opinion, AhR knockout has effects on both systemic and local immunity. Further work is needed to clarify the specific effect and mechanism of AhR to regulate immunity in EAU.

Aryl hydrocarbon receptor signaling has also been identified to play a crucial role in tumor immunity (62). Although several studies have suggested that AhR may be a suppressor of tumor under specified conditions (63), the effects of AhR activation have also been suggested to occupy a significant place in immune modulation and carcinogenesis *in vivo* and *in vitro* (64–66). Thus, it is indeed necessary to objectively understand the role of AhR in different diseases.

In conclusion, this study expands our understanding of how the AhR regulates immune responses in EAU. In addition that AhR influences acquired immunity as previously reported, we provide a novel evidence showing that AhR is a potent regulator of the innate immune response during the development of EAU. TCDD, the best-known AhR agonist, can binds to AhR with the highest affinity (67). TCDD-mediated functions are almost exclusively AhR dependent, as AhR null mice are completely resistant to TCDD (62, 68, 69). Although TCDD was found to effectively inhibit the development of EAU, it cannot be considered for clinical use in view of its high toxicity. Our future studies will focus on more efficient and less toxic substitutes such as other physiological ligands of AhR that can be metabolized by AhR-regulated cytochrome P4501 (CYP1) enzymes. AhR may represent an attractive target for pharmacological intervention in EAU.

## Ethics Statement

All the experimental procedures were carried out conforming to the ARVO statement for the Use of Animals in Ophthalmic and Vision Research. The protocol was approved by the Ethics Committee of the First Affiliated Hospital of Chongqing Medical University (Number: 2016-171).

## Author Contributions

YH, JH, and SH designed the research project. YH and JH performed EAU induction, Evans blue, Western blot, immunofluorescence, TUNEL staining, and are major contributors in writing the manuscript. HL provided AhR knockout mice and helped plan the study. KH helped plan the study. SJ performed H&E staining. LY performed clinical grading. SM performed ELISA. XZ and JY participated in manuscript writing. AK helped English editing and proofread the manuscript. PY and SH reviewed the manuscript. All the authors read and approved the final manuscript.

## Conflict of Interest Statement

The authors declare that the research was conducted in the absence of any commercial or financial relationships that could be construed as a potential conflict of interest.

## Abbreviations

EAU, experimental autoimmune uveitis; BRB, blood–retinal barrier; TNF-α, tumor necrosis factor-α; iNOS, inducible nitric oxide synthase; AhR, aryl hydrocarbon receptor; TCDD, tetrachlorodibenzo-p-dioxin; Arg-1, arginase-1; ZO-1, zonula occludens-1; Bax, Bcl-2 associated X protein; STAT, signal transducers and activators of transcription; GAPDH, glyceraldehyde-3-phosphate dehydrogenase; Iba1, ionized calcium-binding adaptor molecule 1; IOD, integrated optic density; TUNEL, TdT-mediated dUTP nick end labeling; ELISA, enzyme-linked immunosorbent assay; IL, interleukin.
