# Supplementary material for: Aryl Hydrocarbon Receptor Regulates Apoptosis and Inflammation in a Murine Model of Experimental Autoimmune Uveitis
Source: Front Immunol. 2018 Jul 25;9:1713. doi: 10.3389/fimmu.2018.01713 (PMC6068235; doi:10.3389/fimmu.2018.01713)
Supplement: Supplementary file 1 [file image_1.PDF]

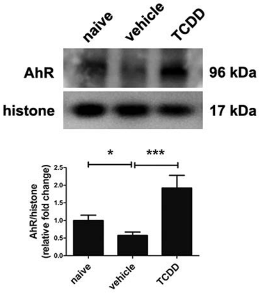


**Supplementary Figure 1.** Western blot followed a standard technique as described in the manuscript. AhR (1:500, Abcam, UK), Histone 3 (1:5000, Proteintech, China) was used as a loading control to nuclear protein. The nuclear protein expression of AhR between naive group, vehicle-treated group and TCDD treated group (n=4/group; mean ± SD; *$p < 0.05$; ***$p < 0.001$; one-way ANOVA).
